# Supplementary material for: Resolving the full spectrum of human genome variation using Linked-Reads
Source: Genome Res. 2019 Apr;29(4):635–45. doi: 10.1101/gr.234443.118 (PMC6442396; doi:10.1101/gr.234443.118)
Supplement: Supplemental Material [file supp_gr.234443.118_Supplemental_longranger-2.2.zip › longranger-2.2.tar.bz2!/lr2.2-public/lib/stan/cmdstan/stan/doc/api/html/math_2functions_2log1m_8hpp_source.html]

Stan: src/stan/math/functions/log1m.hpp Source File


|  |  |
| --- | --- |
|  | Stan  2.5.0  probability, sampling & optimization |


- Main Page
- Related Pages
- Namespaces
- Classes
- Files

- File List
- File Members

All Classes Namespaces Files Functions Variables Typedefs Enumerations Enumerator Friends Macros Pages

- src
- stan
- math
- functions

log1m.hpp

Go to the documentation of this file.

1 #ifndef STAN\_\_MATH\_\_FUNCTIONS\_\_LOG1M\_HPP

2 #define STAN\_\_MATH\_\_FUNCTIONS\_\_LOG1M\_HPP

3

4 #include <boost/math/tools/promotion.hpp>

5 #include <stan/math/functions/log1p.hpp>

6

7 namespace stan {

8  namespace math {

9

38  template <typename T>

39  inline typename boost::math::tools::promote\_args<T>::type

40  log1m(T x) {

41  return log1p(-x);

42  }

43

44  }

45 }

46

47 #endif

stan::math::log1p

boost::math::tools::promote\_args< T >::type log1p(const T x)

Return the natural logarithm of one plus the specified value.

**Definition:** log1p.hpp:39

log1p.hpp

stan::math::log1m

boost::math::tools::promote\_args< T >::type log1m(T x)

Return the natural logarithm of one minus the specified value.

**Definition:** log1m.hpp:40


---

[ Stan Home Page ]

*© 2011–2014,
Stan Development Team.*
